# Sex-dependent intra-islet structural rearrangements affecting alpha-to-beta cell interactions lead to adaptive enhancements of Ca^2+^ dynamics in prediabetic beta cells

**DOI:** 10.1007/s00125-024-06173-w

**Published:** 2024-05-30

**Authors:** Montse Visa, Per-Olof Berggren

**Affiliations:** 1 The Rolf Luft Research Center for Diabetes and Endocrinology, Karolinska Institutet, Stockholm, Sweden; 2 Diabetes Research Institute, University of Miami Miller School of Medicine, Miami, FL USA; 3 Tecnológico de Monterrey, Real San Agustín, Mexico; 4 West China Hospital, Sichuan University, Chengdu, China; 5 School of Biomedical Sciences, Ulster University, Coleraine, UK

**Keywords:** Alpha cell input, Beta cell function, Ca^2+^ imaging, Prediabetes, Sex differences

## Abstract

**Aims/hypothesis:**

Prediabetic pancreatic beta cells can adapt their function to maintain normoglycaemia for a limited period of time, after which diabetes mellitus will manifest upon beta cell exhaustion. Understanding sex-specific beta cell compensatory mechanisms and their failure in prediabetes (impaired glucose tolerance) is crucial for early disease diagnosis and individualised treatment. Our aims were as follows: (1) to determine the key time points of the progression from beta cells’ functional adaptations to their failure in vivo; and (2) to mechanistically explain in vivo sex-specific beta cell compensatory mechanisms and their failure in prediabetes.

**Methods:**

Islets from male and female transgenic *Ins1*^CreERT2^-*GCaMP3* mice were transplanted into the anterior chamber of the eye of 10- to 12-week-old sex-matched C57BL/6J mice. Recipient mice were fed either a control diet (CD) or western diet (WD) for a maximum of 4 months. Metabolic variables were evaluated monthly. Beta cell cytoplasmic free calcium concentration ([Ca^2+^]_i_) dynamics were monitored in vivo longitudinally by image fluorescence of the GCaMP3 reporter islets. Global islet beta cell [Ca^2+^]_i_ dynamics in line with single beta cell [Ca^2+^]_i_ analysis were used for beta cell coordination studies. The glucagon receptor antagonist L-168,049 (4 mmol/l) was applied topically to the transplanted eyes to evaluate in vivo the effect of glucagon on beta cell [Ca^2+^]_i_dynamics. Human islets from non-diabetic women and men were cultured for 24 h in either a control medium or high-fat/high-glucose medium in the presence or absence of the glucagon receptor antagonist L-168,049. [Ca^2+^]_i_ dynamics of human islets were evaluated in vitro after 1 h exposure to Fura-10.

**Results:**

Mice fed a WD for 1 month displayed increased beta cell [Ca^2+^]_i_ dynamics linked to enhanced insulin secretion as a functional compensatory mechanism in prediabetes. Recruitment of inactive beta cells in WD-fed mice explained the improved beta cell function adaptation observed in vivo; this occurred in a sex-specific manner. Mechanistically, this was attributable to an intra-islet structural rearrangement involving alpha cells. These sex-dependent cytoarchitecture reorganisations, observed in both mice and humans, induced enhanced paracrine input from adjacent alpha cells, adjusting the glucose setpoint and amplifying the insulin secretion pathway. When WD feeding was prolonged, female mice maintained the adaptive mechanism due to their intrinsically high proportion of alpha cells. In males, [Ca^2+^]_i_ dynamics progressively declined subsequent to glucose stimulation while insulin secretion continue to increase, suggesting uncoordinated beta cell function as an early sign of diabetes.

**Conclusions/interpretation:**

We identified increased coordination of [Ca^2+^]_i_ dynamics as a beta cell functional adaptation mechanisms in prediabetes. Importantly, we uncovered the mechanisms by which sex-dependent beta cell [Ca^2+^]_i_ dynamics coordination is orchestrated by an intra-islet structure reorganisation increasing the paracrine input from alpha cells on beta cell function. Moreover, we identified reduced [Ca^2+^]_i_ dynamics coordination in response to glucose as an early sign of diabetes preceding beta cell secretory dysfunction, with males being more vulnerable. Alterations in coordination capacity of [Ca^2+^]_i_ dynamics may thus serve as an early marker for beta cell failure in prediabetes.

**Graphical Abstract:**

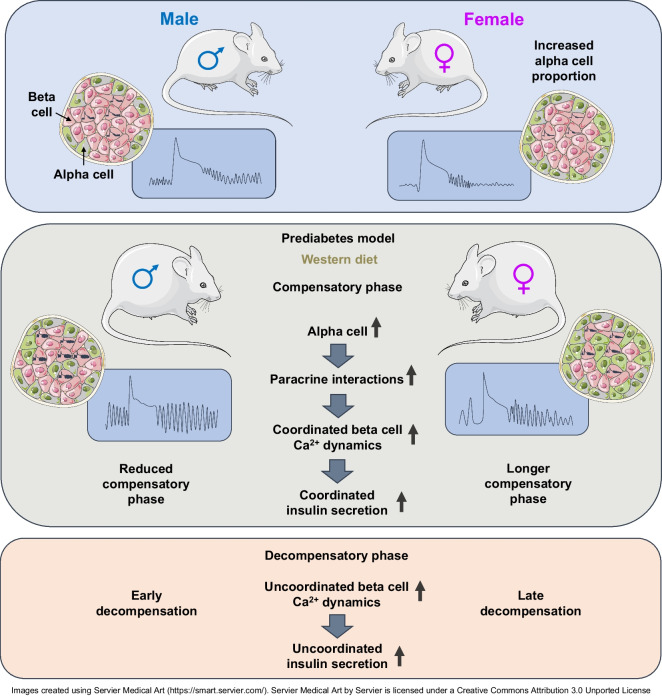

**Supplementary Information:**

The online version of this article (10.1007/s00125-024-06173-w) contains peer-reviewed but unedited supplementary material.


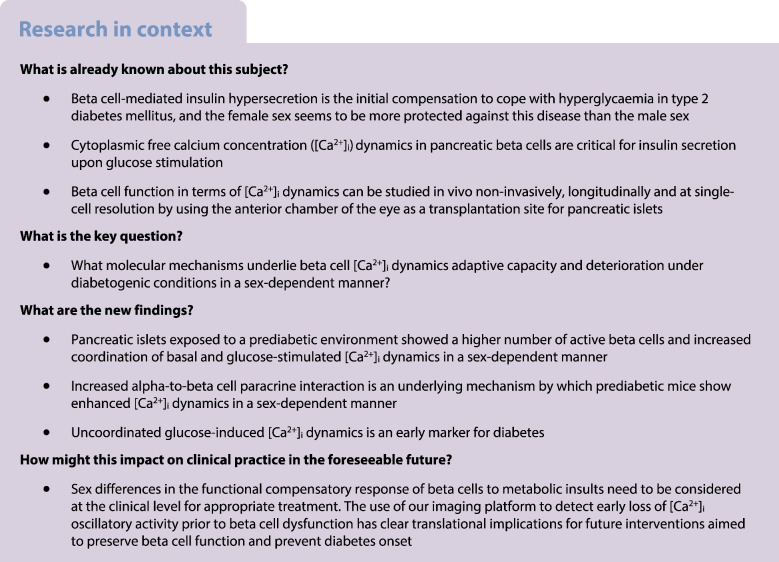


## Introduction

In type 2 diabetes, pancreatic beta cells initially compensate for peripheral insulin resistance by adapting their function and mass to secrete more insulin and retain normoglycaemia [1, 2]. Evidence for a predominant role of beta cell functional adaptation over beta cell mass compensation has been described both in vitro and in vivo in different models of prediabetes (impaired glucose tolerance) [3–6]. However, the molecular mechanisms underlying beta cell functional adaptation are not fully understood. Sex differences in energy metabolism and glucose homeostasis elicit different susceptibility to type 2 diabetes, with higher prevalence in the male sex than in the female sex in both humans and rodents [7]. The protective effect against metabolic disorders shown in fertile females seems to be attributable to the beneficial actions of oestrogens. However, further investigations are needed to dissect the mechanisms involving sex-dimorphic glucose homeostasis regulation and beta cell insulin secretory capacity.

Proper pancreatic beta cell function requires intact glucose sensing, glucose metabolism and insulin secretory machinery [8], in addition to coordinated secretory activity of individual beta cells. Hence, beta cells within the pancreatic islet are connected in three dimensions and this connectivity is enhanced upon glucose stimulation [9, 10]. Moreover, alpha-to-beta cell communication plays a critical role in the regulation of beta cell secretion [11, 12], with glucagon acting as a key regulator of the glycaemic set point [13]. To what extent this paracrine connectivity may adapt to high metabolic demands is not yet clear.

Cytoplasmic free calcium concentration ([Ca^2+^]_i_) dynamics has been used as a read-out of beta cell function [14, 15]. Functional beta cell subpopulations with different [Ca^2+^]_i_ oscillatory patterns have been identified under healthy conditions [14, 16]. In contrast, altered beta cell [Ca^2+^]_i_ dynamics linked to defective insulin secretion and glucose tolerance have been described in animal models of type 2 diabetes [15, 17] and in islets from humans with type 2 diabetes [18]. [Ca^2+^]_i_ dynamics also has an essential role in beta cell pulsatile insulin secretion [19]. Disrupted coordination of beta cell [Ca^2+^]_i_ dynamics has been observed in islets from high-fat-diet-treated mice [20], *ob/ob* mice [21] and islets from individuals with type 2 diabetes [10]. However, little is known about the beta cell [Ca^2+^]_i_ compensatory mechanisms taking place in early stages of prediabetes.

Previous studies have mostly been done in vitro where vascularisation, innervation and cell-to-cell communication are disrupted. Hence, key factors known to contribute to coordination of beta cell [Ca^2+^]_i_ dynamics [22, 23] are compromised. Using the anterior chamber of the eye (ACE) as a transplantation site has enabled detailed in vivo studies of beta cell [Ca^2+^]_i_ dynamics to be carried out non-invasively, longitudinally and at single-cell resolution [24]. Applying this approach, we have investigated in vivo the pattern of beta cell [Ca^2+^]_i_ dynamics in beta cell functional adaptation in both sexes of a model of diet-induced type 2 diabetes, going from prediabetes to manifest diabetes. Moreover, we have addressed alpha-to-beta cell communication within the pancreatic islet as a molecular mechanism underlying beta cell [Ca^2+^]_i_ adaptation.

## Methods

### Mice and diets

For in vivo beta cell Ca^2+^ imaging studies, pancreatic islets from a mouse model expressing the Ca^2+^ biosensor GCaMP3 in beta cells (*Ins1*^CreERT2^*-GCaMP3*) were used. *Ins1*^CreERT2^*-GCaMP3* mice were generated by crossing B6(Cg)-*Ins1*^*tm2.1(cre/ERT2)Thor*^/J (The Jackson Laboratories, USA; https://www.jax.org/strain/026802; RRID:IMSR_JAX:026802) [25]) with B6;129S-*Gt(ROSA)26Sor*^*tm38(CAG−GCaMP3)Hze*^/J (The Jackson Laboratories; https://www.jax.org/strain/014538; RRID:IMSR_JAX:014538) [26]) mice. *Ins1*^CreERT2^*-GCaMP3* mice were kept on C57BL/6J background and backcrossed at the animal core facility at Karolinska Institutet. Beta cell-specific GCaMP3 expression was induced in 8- to 10-week-old *Ins1*^CreERT2^*-GCaMP3* mice by s.c. injections of tamoxifen (2 mg twice per week for 2 weeks) to reach 60–70% beta cell recombination [25]. For diet studies, C57BL/6J male and female mice (Charles River, MA, USA) were fed with control diet (CD; 11% energy from fat, Ssniff Spezialdiäten Germany) or western diet (WD; 43% energy from fat, Ssniff Spezialdiäten) for up to 4 months. The diet intervention was performed twice in independent cohorts of mice with at least four mice for each experimental group. Littermates from the same sex were housed in groups of four or five animals per cage on a 12 h light–dark cycle in temperature- and humidity-controlled environment with ad libitum access to food. All experiments were performed in accordance with the guidelines for care and use of animals in research at Karolinska Institutet and were approved by the animal ethics committee.

### Pancreatic islet isolation and transplantation

Islets were isolated from 10- to 12-week-old tamoxifen-induced *Ins1*^CreERT2^*-GCaMP3* male and female mice by collagenase digestion and islet handpicking [24]. Prior to transplantation, islets were allowed to recover for 24 h in RPMI with 11 mmol/l glucose. Six to ten islets were transplanted into the ACE of 8- to 10-week-old C57BL/6J female and male mice [24]. The sexes of donor and recipient mice were matched (male-to-male, female-to-female).

### In vivo beta cell [Ca^2+^]_i_ imaging

In vivo beta cell [Ca^2+^]_i_ dynamics imaging was performed 3 months after transplantation and once a month until the end of the diet treatment. For longitudinal studies, the same islet was imaged over time. Mice fasted for 4 h were anaesthetised using fluanisone/fentanyl/midazolam (20/0.6/10 mg/kg). The GCaMP3 fluorescence signal of the islets was collected for 10 min in the non-stimulated state and then 40 min after tail vein glucose injection (0.4 g/kg). The glucagon receptor antagonist L-168,049 (4 mmol/l, Tocris) was topically applied to the eye and islets were imaged for 20 min (see electronic supplementary material [ESM] Methods for details).

### [Ca^2+^]_i_ image processing and analysis

In vivo beta cell [Ca^2+^]_i_ traces were extracted from GCaMP3 fluorescence images processed using custom-made MATLAB scripts [24, 27]. The entire GCaMP3 fluorescence recording for every single cell was normalised against their basal GCaMP3 signal and expressed as fold increase over the baseline (Δ*F*/*F*_0_). Normalised GCaMP3 traces were used to calculate the percentage of cells presenting significant [Ca^2+^]_i_ spiking activity [24, 27], to analyse by power spectral analysis fast (6–60 s) and slow [Ca^2+^]_i_ oscillations (60–600 s) [28] and to evaluate the percentage of glucose-responding beta cells. The term [Ca^2+^]_i_ dynamics was defined as all changes in [Ca^2+^]_i_ including fast and slow oscillations, peak values and plateau values (see ESM Methods for details).

### Beta cell coordination and three-dimensional functional networks

Normalised GCaMP3 traces were used to evaluate similarities of [Ca^2+^]_i_ dynamics within the beta cell pool as an indicator of beta cell coordination. Using a custom-made MATLAB script, Pearson’s correlation analysis was performed in all possible beta cell combinations in an islet, excluding cell autocorrelation. Global islet beta cell [Ca^2+^]_i_ dynamics coordination was calculated as mean of all positive beta cell pair coefficients of correlation within an islet (mean *r*). Cell pairs correlated >0.8 were used to build three-dimensional (3D) functional beta cell networks, using the Cartesian coordinates in the *x*, *y*, *z* planes and line connectors between highly correlated beta cell pairs.

### Metabolic studies

IPGTT and IPITT were performed monthly in mice fasted for 6 h. Blood glucose concentrations were measured at basal state (0 min) and at the indicated time points after i.p. injection of glucose (2 g/kg; IPGTT) or insulin (0.5 U/kg; IPITT). IVGTT was performed in mice fasted for 4 h and anaesthetised with fluanisone / fentanyl / midazolam (20/0.6/10 mg/kg). Blood glucose was measured at basal state (0 min) and at the indicated time points after glucose injection (0.4 g/kg) through the tail vein. Plasma insulin levels were measured using an AlphaLISA kit (Perkin Elmer) and glucagon levels using an ELISA kit (Crystal Chem).

### Human islet culture and treatment

Human islets from non-diabetic women and men were obtained from the Nordic Network for Islet Transplantation (see ESM Table 1 and ESM Human islets checklist for details). Data on the sex of participants was provided by the Nordic Islet Computer System database. Islets were cultured in CMRL1066 medium with 5.5 mmol/l glucose (control) or with 300 μmol/l palmitate and 15 mmol/l glucose (high-fat/high-glucose [HFHG]) for 24 h in the presence or absence of the glucagon receptor antagonist L-168,049 (50 nmol/l).

### In vitro [Ca^2+^]_i_ measurement

Human islets were loaded for 1 h with Fura-10 (2 μmol/l; AATBioquest) in a buffered solution at 3 mmol/l glucose. [Ca^2+^]_i_ signal was recorded in islets perifused at 3 mmol/l or 11 mmol/l glucose in the presence or absence of the glucagon receptor antagonist L-168,049 (50 nmol/l). Fluorescence values (*F*) were expressed as the ratio of fluorescence at 354 and 415 nm (*F*_354_/*F*_415_). [Ca^2+^]_i_ levels at 3 mmol/l glucose (*F*_0_), first stimulatory [Ca^2+^]_i_ peak after perifusion at 11 mmol/l glucose (Δ*F*−*F*_0_) and amplitude of slow [Ca^2+^]_i_ oscillations were analysed (see ESM Methods for details).

### Immunostaining and islet morphometric analysis

Pancreases, explanted islets from the ACE and islets in culture were collected and immunostained for insulin and glucagon (see ESM Methods for details). Mantel and core areas of the islets were analysed, differentiating the area 20 µm from the external islet perimeter for mantel area quantification and the rest of the islet for core quantification [29].

### Statistical analysis

Data are presented as individual points or mean ± SEM. Statistical differences were analysed by Student’s *t* test or by one- or two-way ANOVA, with Tukey and Bonferroni post hoc test, using GraphPad Prism (version 8.4.1; https://www.graphpad.com/) and MATLAB (version R2017a; https://www.mathworks.com). Differences were considered significant at *p* values <0.05.

## Results

### In vivo adaptation of beta cell [Ca^2+^]_i_ dynamics in a WD-induced prediabetes model

**Fig. 1:**
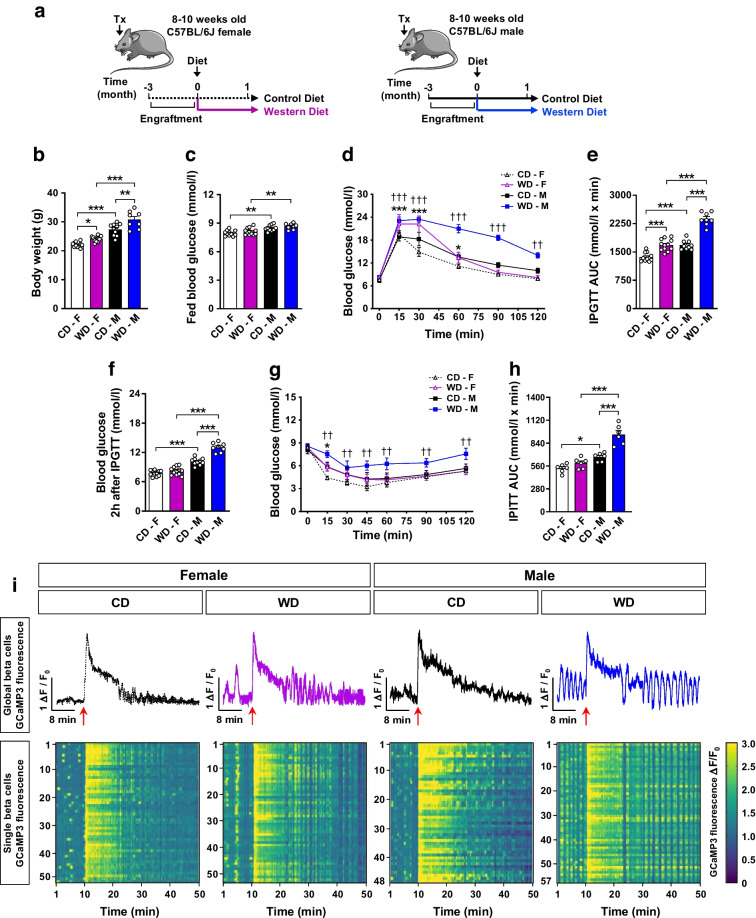
Changes in in vivo beta cell [Ca^2+^]_i_ dynamics and glucose homeostasis after 1 month on WD. C57Bl/6J female (F) and male (M) mice were transplanted (Tx) with *Ins1*^CreERT2^-*GCaMP3* reporter islets into the ACE and fed a CD or WD for 1 month. (**a**) Schematic illustration of the experimental timeline. This schematic was created using Servier Medical Art (https://smart.servier.com/). Servier Medical Art by Servier is licensed under a Creative Commons Attribution 3.0 Unported License. (**b**, **c**) Body weight (**b**) and non-fasting blood glucose levels (**c**) after 1 month of diet (*n*=8–12 mice/group). (**d**, **e**) IPGTT blood glucose levels (**d**) and corresponding AUC (**e**) after 1 month of diet (*n*=8–12 mice/group). (**f**) Blood glucose levels 2 h after IPGTT (*n*=8–12 mice/group). (**g**, **h**) IPITT blood glucose levels (**g**) and corresponding AUC (**h**) after 1 month of diet (*n*=6 mice/group). (**i**) Representative GCaMP3 fluorescent signal (Δ*F*/*F*_0_) of *Ins1*^CreERT2^-*GCaMP3* transplanted islets after 1 month diet. Single beta cell GCaMP3 fluorescence intensities corresponding to the global GCaMP3 traces shown are presented as heat maps, with the colour code denoting normalised GCaMP3 fluorescence intensity. Red arrows indicate the time point when glucose was injected intravenously. Data correspond to two independent experiments with at least four mice for each experimental group. Data are presented as individual points (**b**, **c**, **e**, **f**, **h**) or mean ± SEM (**d**, **g**). Statistics are based on one-way ANOVA (**b**, **c**, **e**, **f**, **h**; *p<0.05, **p<0.01, ***p<0.001) or two-way ANOVA (**d**, **g**; *p<0.05, ***p<0.001 WD-F vs CD-F; ^††^*p*<0.01, ^†††^*p*<0.001 WD-M vs CD-M)

To investigate in vivo sex-specific [Ca^2+^]_i_ compensatory mechanisms in the beta cell functional adaptive phase prior to diabetes onset, we used a WD-induced mouse model of type 2 diabetes. In 8- to 10-week-old C57BL/6J female and male mice, we transplanted islets from *Ins1*^CreERT2^-*GCaMP3* sex-matched donor mice into the ACE. Three months after transplantation, mice of the same sex were randomly divided into two groups with similar metabolic features (ESM Fig. 1a–e) and assigned to CD or WD (Fig. 1a). After 1 month on diet, WD-fed mice showed increased body weight (Fig. 1b), with no changes in fed blood glucose (Fig. 1c) but impaired glucose tolerance (Fig. 1d,e) compared with their respective sex-matched CD-fed groups. Interestingly, male mice on WD showed increased body weight gain (Fig. 1b) and worsen glycaemic control than WD-fed females (Fig. 1d–f). Moreover, male mice on WD clearly exhibited insulin resistance (Fig. 1g,h), while WD-fed females only showed significantly higher glucose levels 15 min after insulin administration. Hence, female mice presented a more resilient metabolic phenotype to WD exposure than male mice.

WD-induced glucose tolerance differences between sexes were confirmed under the in vivo [Ca^2+^]_i_ dynamics protocol, in which mice were anaesthetised and intravenously stimulated with glucose (ESM Fig. 2a–c). In vivo [Ca^2+^]_i_ dynamics of transplanted islets demonstrated that mice fed a WD for 1 month exhibited overall increased beta cell [Ca^2+^]_i_ dynamics compared with their respective sex-matched CD groups (Fig. 1i). The GCaMP3 signal of single beta cells showed greater [Ca^2+^]_i_ dynamics variability in CD-fed groups prior to glucose stimulation while the WD-fed groups presented greater beta cell signal consistency (Fig. 1i). After glucose stimulation, the WD-fed mice presented more regular and sustained enhanced [Ca^2+^]_i_ dynamics; this was more evident in the WD-fed male group. These changes were independent of blood glucose differences between sex-matched WD- and CD-fed groups, which exhibited similar blood glucose levels before glucose stimulation and after the imaging sessions (ESM Fig. 2d). Hence, as a compensatory mechanism in prediabetes, beta cells enhance [Ca^2+^]_i_ dynamics both under basal conditions and after glucose stimulation following 1 month of WD feeding.

### WD increases basal [Ca^2+^]_i_ dynamics and coordination of beta cells leading to fasting hyperinsulinaemia

**Fig. 2:**
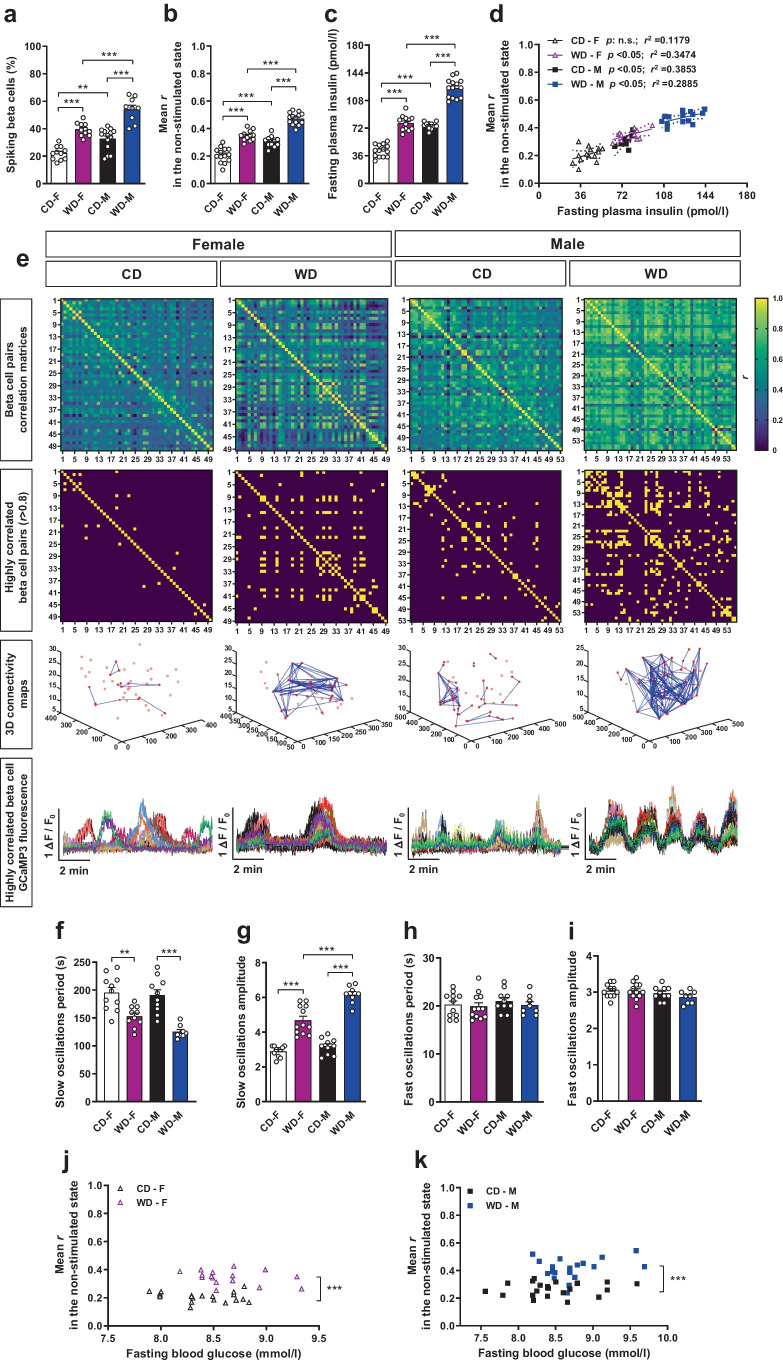
Beta cells from mice exposed to WD enhance basal [Ca^2+^]_i_ dynamics linked to an increase in fasting insulin release by a compensatory mechanism involving improved glucose sensing. *Ins1*^CreERT2^-*GCaMP3* mouse islets transplanted into the ACE of female (F) and male (M) mice fed a CD or WD for 1 month were imaged in vivo at single beta cell resolution under fasting conditions for a period of 10 min. (**a**) Percentage of beta cells presenting significant [Ca^2+^]_i_ spiking activity in relation to global islet GCaMP3 fluorescence (*n*=48–97 beta cells/islet, *n*=8–12 mice/group). (**b**) Mean of the coefficients of correlation (mean *r*) for the GCaMP3 fluorescence signal from all possible combinations of single beta cell pairs (*n*=48–97 beta cells/islet, *n*=11–14 mice/group). (**c**) Fasting plasma insulin levels measured prior to imaging in anaesthetised mice (*n*=11–14 mice/group). (**d**) Mean *r* for beta cell pairs GCaMP3 fluorescence signal in (**b**) vs fasted plasma insulin levels in **c** for every mouse (*n*=11–14 mice/group). Linear regression with a CI of 95% is shown for every experimental group. *p* value and coefficient of determination (*r*^2^) are shown. (**e**) Representative heat maps for every experimental group show coefficient of correlation values for each beta cell pair GCaMP3 fluorescence signal, with colour code indicating coefficient of correlation values (*r*). The correspondent heat maps for highly correlated beta cell pairs (*r*>0.8) are shown with their respective 3D Cartesian maps and the single beta cell GCaMP3 fluorescence traces corresponding to the highly correlated beta cell pairs. (**f**–**i**) Mean period (**f**, **h**) and amplitude (**g**, **i**) of slow (**f**, **g**) and fast oscillations (**h**, **i**) of islets calculated by power spectral analysis of GCaMP3 fluorescence traces from single beta cells, represented as mean of all beta cells in an islet (*n*=48–97 beta cells/islet, *n*=8–12 mice/group). (**j**, **k**) Mean* r* for the GCaMP3 fluorescence signal within an islet vs fasted blood glucose levels measured prior to imaging in anaesthetised mice for every mouse in F (**j**) and M (**k**) groups (*n*=16–23 mice/group). Data correspond to two independent experiments with at least four mice for each experimental group. Data are presented as individual points. Statistics are based on one-way ANOVA; **p<0.01, ***p<0.001

We evaluated beta cell [Ca^2+^]_i_ dynamics in the non-stimulated state to clarify whether the observed changes in basal [Ca^2+^]_i_ dynamics were linked to a compensatory functional beta cell adaptation mechanism. WD-fed groups of mice presented a higher percentage of beta cells showing significant [Ca^2+^]_i_ spiking activity (Fig. 2a), with increased [Ca^2+^]_i_ dynamics coordination (Fig. 2b) and significantly higher fasting plasma insulin levels (Fig. 2c). Interestingly, the correlation of [Ca^2+^]_i_ coordination values with insulin release for each individual mouse demonstrated a positive pattern in all groups (Fig. 2d). Presentations of the coefficients of correlation for each beta cell pair as a heat-map showed that WD-fed mice had an increased number of highly correlated beta cell pairs (Fig. 2e). 3D connectivity maps of highly correlated beta cells showed different degrees of beta cell coordination in CD-fed mice, with few cells involved and located relatively close within the islet (Fig. 2e and ESM Fig. 3a, b). WD feeding induced more complex coordination networks, involving more cells positioned distant within the islet (Fig. 2e and ESM Fig. 3a, b), especially in WD-fed male mice. Accordingly, while [Ca^2+^]_i_ dynamics traces for highly correlated beta cells exhibited different patterns in CD-fed mice (Fig. 2e), they were more consistent under WD feeding. Moreover, WD-fed groups presented reduced period (Fig. 2f) and higher amplitude (Fig. 2g) of the slow oscillations while values for fast oscillations were similar (Fig. 2h,i). Interestingly, the amplitude for slow [Ca^2+^]_i_ oscillations was higher in WD-fed male mice, indicating that males require higher basal [Ca^2+^]_i_ dynamics adaptation than females to counteract an increased metabolic demand. Together, these data demonstrate greater beta cell functional variability under CD conditions while WD feeding induced higher spatiotemporal beta cell [Ca^2+^]_i_ dynamics coordination, especially in WD-fed males. Hence, we observed that under high metabolic demand beta cells were able to increase their basal [Ca^2+^]_i_ dynamics in a coordinated way linked to enhanced controlled insulin secretion.

Moreover, we observed that at comparable blood glucose levels WD-fed mice exhibited higher [Ca^2+^]_i_ dynamics coordination compared with their respective sex-matched CD groups, both in females (Fig. 2j) and in males (Fig. 2k). Thus, the increased [Ca^2+^]_i_ dynamics coordination observed under WD exposure was not attributable to higher blood glucose levels in the WD-fed groups. These data suggest increased beta cell glucose sensing as a trigger for the observed [Ca^2+^]_i_ dynamics adaptation upon WD feeding.

### One month of WD exposure increases beta cell glucose-stimulated [Ca^2+^]_i_ dynamics and insulin secretory activity

**Fig. 3:**
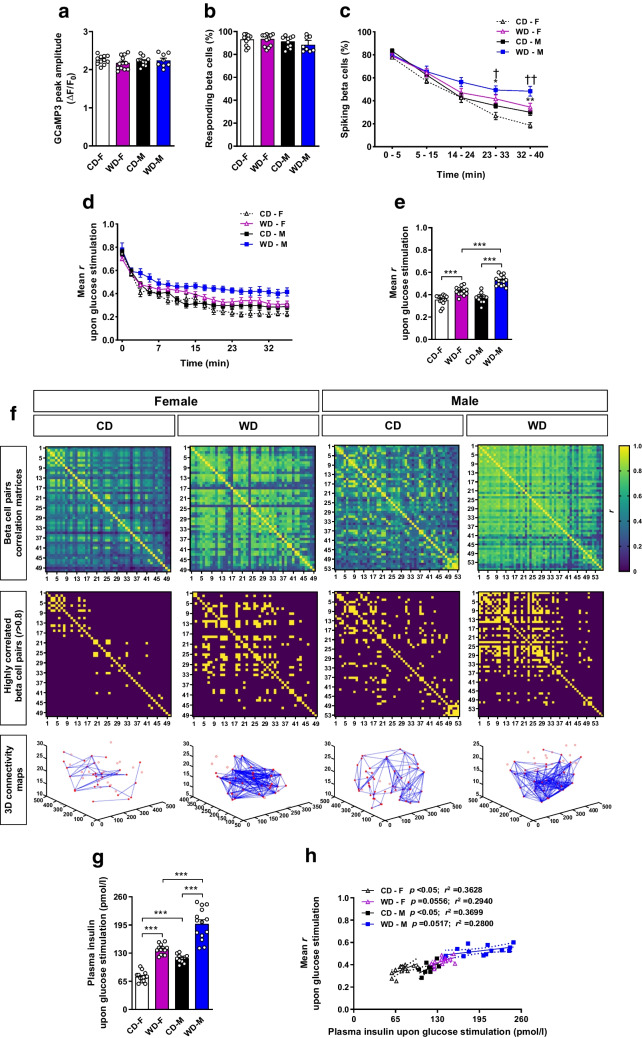
In vivo beta cell insulin secretory capacity in response to glucose is increased by enhanced [Ca^2+^]_i_ dynamics after 1 month of WD. In vivo imaging of *Ins1*^CreERT2^-*GCaMP3* biosensor islets transplanted into the ACE of female (F) and male (M) mice fed a CD or WD for 1 month were analysed at single beta cell resolution after glucose stimulation via tail vein injection. (**a**) Mean amplitude of the first peak detected after glucose stimulation of all beta cells in an islet. (*n*=48–97 beta cells/islet, *n*=8–12 mice/group). (**b**) Percentage of glucose-responding beta cells in an islet (*n*=48–97 beta cells/islet, *n*=8–12 mice/group). (**c**) Percentage of beta cells presenting significant [Ca^2+^]_i_ spiking activity in relation to the global islet GCaMP3 fluorescence analysed for every specified time window after glucose stimulation (*n*=48–97 beta cells/islet, *n*=8–12 mice/group). (**d**) Mean of the coefficients of correlation (mean *r*) for the GCaMP3 fluorescence signal from all possible combinations of single beta cell pairs calculated in the specified time window after glucose challenge (*n*=48–97 beta cells/islet, *n*=11–14 mice/group). (**e**) Mean *r* for the GCaMP3 fluorescence signalling from all possible combinations of single beta cell pairs in the glucose stimulatory period between 5 and 15 min (*n*=48–97 beta cells/islet, *n*=11–14 mice/group). (**f**) Representative heat maps for each experimental group showing the coefficient of correlation values (*r*) for the GCaMP3 fluorescence signal of all possible beta cell pair combinations in the period between 5 and 15 min after glucose stimulation, with the colour code indicating the *r* values. The corresponding heat maps for highly correlated beta cell pairs (*r* >0.8) are shown with their respective 3D Cartesian maps. (**g**) Plasma insulin levels measured after glucose stimulation (10 min after glucose injection) in anaesthetised mice designated for in vivo imaging (*n*=11–14 mice/group). (**h**) Mean *r* for the GCaMP3 fluorescence signal within an islet in the glucose-stimulated state vs glucose-stimulated plasma insulin levels for each individual mouse (*n*=11–14 mice/group). Linear regression with a CI of 95% is shown for every experimental group. *p* value and coefficient of determination (*r*^2^) are shown. Data correspond to two independent experiments with at least four mice for each experimental group. Data are presented as individual points (**a**, **b**, **e**, **g**, **h**) or mean ± SEM (**c**, **d**). Statistics are based on one-way ANOVA (**a**, **b**, **e**, **g**, **h**; *p<0.05, **p<0.01, ***p<0.001) or two-way ANOVA (**c**, **d**; *p<0.05, **p<0.01 WD-F vs CD-F; ^†^*p*<0.05, ^††^*p*<0.01 WD-M vs CD-M)

We next investigated whether beta cell glucose response capacity was retained in WD-fed mice and whether beta cells could further adapt [Ca^2+^]_i_ dynamics upon glucose stimulation. WD-fed and CD-fed groups presented similar values for the first stimulatory [Ca^2+^]_i_ peak (Fig. 3a) and the percentage of glucose-responding beta cells (Fig. 3b), without differences between sexes. Hence, beta cell glucose responsiveness was not compromised after 1 month of WD exposure. Following glucose injection, both male and female WD-fed mice maintained higher [Ca^2+^]_i_ spiking activity (Fig. 3c) and increased [Ca^2+^]_i_ dynamics coordination (Fig. 3d) compared with their sex-matched CD-fed groups until the end of the imaging recording. In the glucose stimulatory period, [Ca^2+^]_i_ dynamics coordination was increased in the WD-fed groups (Fig. 3e), with high numbers of highly coordinated beta cell pairs (Fig. 3f) and enhanced plasma insulin levels compared with their respective CD sex-matched groups (Fig. 3g). When insulin levels were compared with [Ca^2+^]_i_ dynamics coordination, a positive pattern in all groups was observed (Fig. 3h). This demonstrated that in response to a glucose challenge beta cells from WD-fed mice were still able to increase [Ca^2+^]_i_ dynamics coordination linked to insulin secretion regardless of the basal hyperactivated state. However, under WD exposure, female mice required a smaller increase in [Ca^2+^]_i_ coordination than male mice to achieve the same increase in insulin secretion, when either group was compared with its respective CD-fed matched group. Thus, female mice exposed to WD adapted their [Ca^2+^]_i_ activity to increase insulin secretion in a more effective way than WD-fed male mice.

### Female mice maintain basal [Ca^2+^]_i_ dynamics adaptation linked to enhanced insulin secretion for a longer period of time upon prolonged exposure to WD

**Fig. 4:**
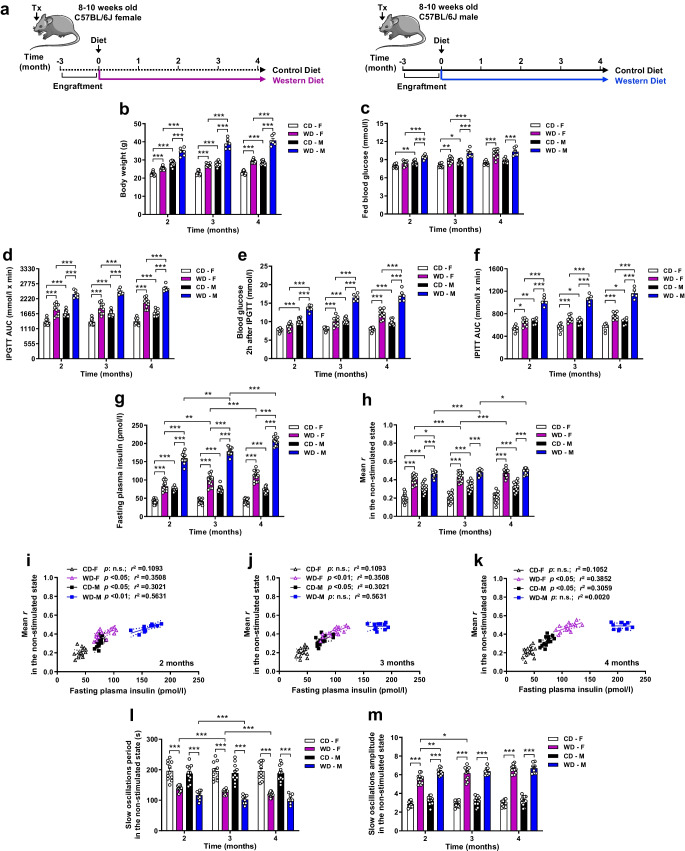
Female mice maintain increased basal [Ca^2+^]_i_ dynamics linked to enhanced insulin secretion for a longer period of time than males over the course of 4 months of WD exposure. C57Bl/6J female (F) and male (M) mice were transplanted (Tx) with *Ins1*^CreERT2^-*GCaMP3* islets into the ACE and fed CD or WD for the indicated periods of time. Transplanted islets were imaged in the non-stimulated state for 10 min at the indicated time points after diet exposure. (**a**) Schematic illustration of the experimental timeline. This schematic was created using Servier Medical Art (https://smart.servier.com/). Servier Medical Art by Servier is licensed under a Creative Commons Attribution 3.0 Unported License. (**b**, **c**) Body weight (**b**) and non-fasting blood glucose levels (**c**) during the study (*n*=6–10 mice/group). (**d**, **e**) IPGTT blood glucose AUC (**d**) and blood glucose levels 2 h after glucose injection in the IPGTT (**e**) (*n*=6–10 mice/group). (**f**) IPITT blood glucose AUC during the study (*n*=5–8 mice/group). (**g**) Fasting plasma insulin levels measured during the study (*n*=11–14 mice/group). (**h**) Mean of the coefficients of correlation (mean *r*) in the non-stimulated state for single beta cell pairs GCaMP3 fluorescence traces from transplanted islets imaged longitudinally along the study (*n*=48–97 beta cells/islet, *n*=11–14 mice/group). (**i**–**k**) Mean *r* for beta cell pairs GCaMP3 fluorescence signal during basal state vs fasted plasma insulin levels for every individual mouse at 2 months (**i**), 3 months (**j**) or 4 months (**k**) of WD exposure (*n*=11–14 mice/group). Linear regression with a CI of 95% is shown for every experimental group. *p* value and coefficient of determination (*r*^2^) are shown. (**l**, **m**) Mean period (**l**) and amplitude (**m**) of slow oscillations of an islet calculated by power spectral analysis of GCaMP3 fluorescence traces in single beta cells and represented as mean of all beta cells in an islet (*n*=48–97 beta cells/islet, *n*=8–11 mice/group). Data correspond to two independent experiments with at least four mice for each experimental group. Data are presented as individual points. Statistics are based on one-way ANOVA; *p<0.05, **p<0.01, ***p<0.001

To evaluate whether the beta cell [Ca^2+^]_i_ dynamics adaptive mechanisms linked to increased insulin secretory capacity observed after 1 month WD exposure persist over time, we performed longitudinal in vivo [Ca^2+^]_i_ dynamics imaging of the same islets from *Ins1*^CreERT2^-*GCaMP3* female and male mice transplanted into the ACE of sex-matched C57Bl6J mice that were then fed a CD or WD for 4 months (Fig. 4a). Sustained WD feeding induced faster metabolic deterioration in male mice than in female mice. WD-fed males exhibited more pronounced body weight increase (Fig. 4b), faster onset of fed hyperglycaemia (Fig. 4c), worse glucose tolerance (Fig. 4d,e) and faster development of insulin resistance (Fig. 4f) when compared with WD-fed female mice. While basal plasma insulin levels continuously increased for both sexes (Fig. 4g), basal [Ca^2+^]_i_ dynamics coordination increased constantly in WD-fed female mice whereas males fed a WD presented a smaller increase at the end of the diet intervention period (Fig. 4h). The correlation of insulin values with [Ca^2+^]_i_ dynamics coordination revealed a positive trend in WD-fed female mice during the entire diet intervention period (Fig. 4i–k) while WD-fed males only retained this adaptive mechanism for 2 months (Fig. 4i). From 3 months of WD onwards, the hyperinsulinaemia in WD-fed male mice was not correlated with increased beta cell [Ca^2+^]_i_ dynamics coordination (Fig. 4j,k). The analysis of the period (Fig. 4l) and amplitude (Fig. 4m) for slow oscillations demonstrated that female mice were capable of adapting both parameters for a longer period of time than males under WD exposure. All together these data indicates that male mice reach their maximum adaptive capacity for [Ca^2+^]_i_ dynamics coordination faster than females in the non-stimulated state while still hypersecreting insulin, indicating uncoordinated and uncontrolled basal insulin hypersecretion.

### Prolonged exposure to WD progressively impairs glucose-stimulated [Ca^2+^]_i_ dynamics in a sex-specific manner

**Fig. 5:**
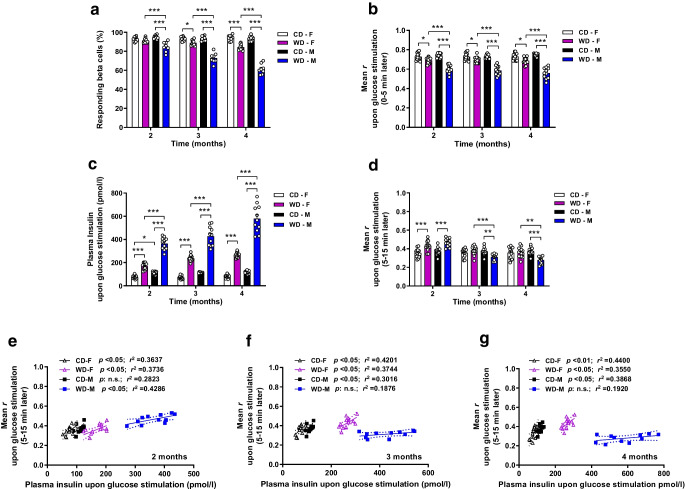
Adaptation upon glucose stimulation is disturbed faster in male mice than in female mice exposed to sustained WD feeding. *Ins1*^CreERT2^-*GCaMP3* islets transplanted into the ACE of C57Bl/6J female (F) and male (M) mice were imaged after glucose stimulation via tail vein injection at the indicated time points after CD or WD exposure. (**a**) Percentage of glucose-responding beta cells assessed longitudinally along the study in the same transplanted islets (*n*=48–97 beta cells/islet, *n*=8–10 mice/group). (**b**) Mean of the coefficients of correlation (mean *r*) for the GCaMP3 fluorescence in single beta cell pairs measured in the same islets along the study and assessed in the stimulatory period after glucose injection (0–5 min) (*n*=48–97 beta cells/islet, *n*=11–14 mice/group). (**c**) Plasma insulin levels measured during the stimulatory period (10 min after glucose injection) longitudinally along the study (*n*=11–14 mice/group). (**d**) Mean *r* for the GCaMP3 fluorescence in single beta cell pairs calculated along the study and assessed in the time window with enhanced [Ca^2+^]_i_ dynamics after the initial stimulatory peak (5–15 min) (*n*=48–97 beta cells/islet, *n*=11–14 mice/group). (**e**–**g**) Mean *r* for the GCaMP3 fluorescence signal within an islet in the glucose-stimulated state vs glucose-stimulated plasma insulin levels for each individual mouse at 2 months (**e**), 3 months (**f**) or 4 months (**g**) of WD exposure (*n*=11–14 mice/group). Linear regression with a CI of 95% is shown for every experimental group. *p* value and coefficient of determination (*r*^2^) are shown. Data correspond to two independent experiments with at least four mice for each experimental group. Data are presented as individual points. Statistics are based on one-way ANOVA; *p<0.05, **p<0.01, ***p<0.001

Sex differences also governed the adaptive capacity of beta cells to glucose-stimulated [Ca^2+^]_i_ dynamics. WD-fed male mice exhibited a faster decrease in the percentage of responding cells over time while females only exhibited a significant reduction after 3 months of exposure to WD (Fig. 5a). Accordingly, beta cell [Ca^2+^]_i_ dynamics coordination during the first phase of glucose stimulation was strongly reduced in the WD-fed group of male mice, whereas females fed a WD exhibited a less marked reduction over time (Fig. 5b). Thus, the effect of WD on glucose response capacity was more deleterious in males than in females. Stimulated plasma insulin levels increased progressively over time in both WD-fed groups, with substantially higher levels observed in males (Fig. 5c). However, [Ca^2+^]_i_ dynamics coordination for the glucose stimulatory period was maintained during the whole diet intervention in WD-fed females, while in males fed a WD a decline in the coordination was observed from the third month onwards (Fig. 5d). The correlation of stimulated insulin levels with [Ca^2+^]_i_ dynamics coordination demonstrated a loss of linearity between these two variables in the WD-fed male group from 3 months onwards while females fed a WD retained a positive correlation throughout the diet intervention (Fig. 5e–g). These data indicate that upon glucose stimulation female mice are able to adapt [Ca^2+^]_i_ dynamics coordination linked to insulin secretion for a longer period of time than male mice.

### Paracrine alpha cell input contributes to beta cell [Ca^2+^]_i_ dynamics functional adaptation during WD feeding

**Fig. 6:**
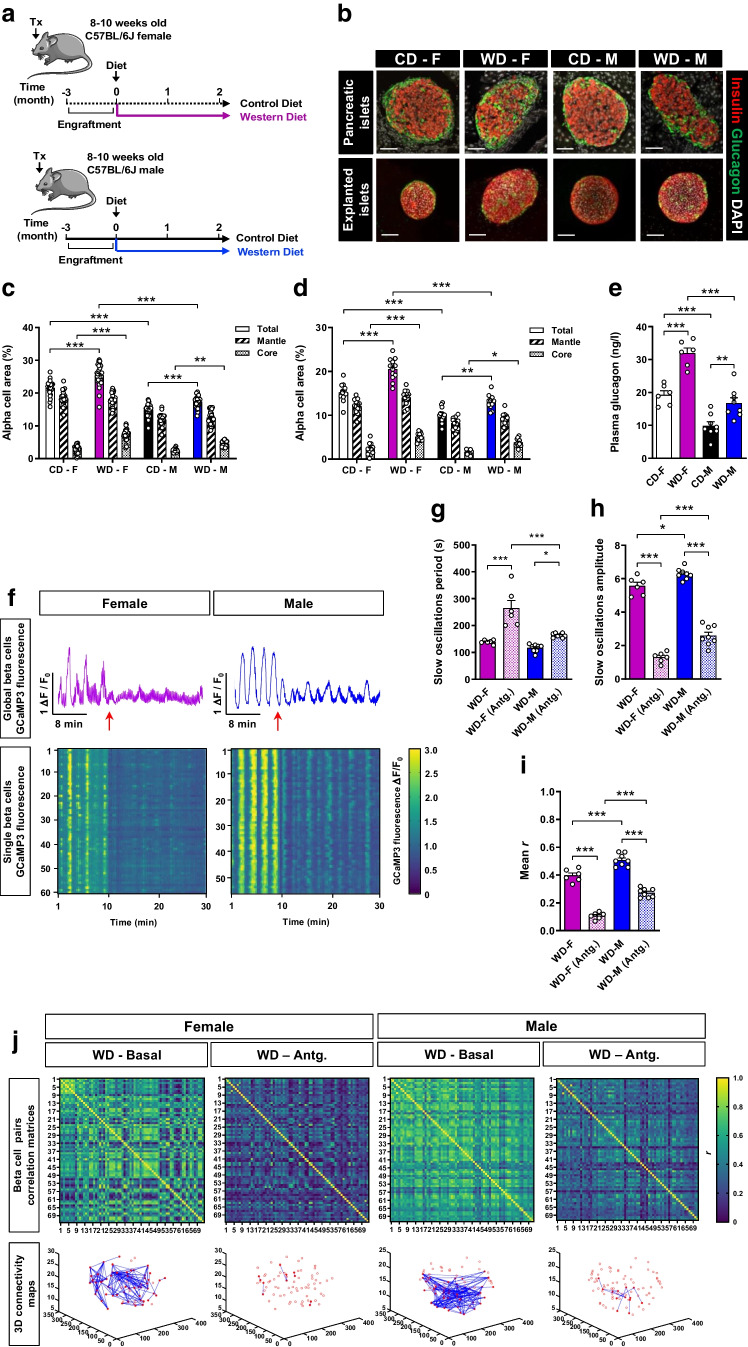
Alpha cell input contributes to the enhanced [Ca^2+^]_i_ dynamics observed under WD feeding. C57Bl/6J female (F) and male (M) mice were transplanted (Tx) with *Ins1*^CreERT2^-*GCaMP3* islets into the ACE and fed a CD or WD for 2 months. In vivo imaging of transplanted islets was analysed at single beta cell resolution after topical administration of the glucagon receptor antagonist L-168,049 (Antg.). (**a**) Schematic illustration of the experimental timeline. This schematic was created using Servier Medical Art (https://smart.servier.com/). Servier Medical Art by Servier is licensed under a Creative Commons Attribution 3.0 Unported License. (**b**) Representative confocal images of immunolabelled pancreatic sections and explanted islets showing immunostaining for beta cells by insulin (red), alpha cells by glucagon (green) and nuclei by DAPI (white). Scale bar, 50 μm. (**c**) Total alpha cell area and differentiating mantle and core regions of the islet expressed as % of total islet area and measured in all islets detected in three different sections per pancreas (*n*=3 mice/group). (**d**) Same analysis as (**c**) measured in islets explanted from the ACE (*n*=11–13 islets/group). (**e**) Fasting plasma glucagon levels (*n*=6–8 mice/group). (**f**) Representative normalised global beta cell GCaMP3 fluorescence (Δ*F*/*F*_0_) for an islet and the corresponding individual beta cell GCaMP3 fluorescence traces represented as heat maps with colour code denoting normalised GCaMP3 fluorescence intensity. Red arrows indicate the time point at which the glucagon receptor antagonist L-168,049 was topically applied (10 min after start of the imaging period). (**g**, **h**) Period (**g**) and amplitude (**h**) of slow oscillations from the GCaMP3 fluorescence traces analysed by power spectral analysis in single beta cells before and after the application of the glucagon receptor antagonist (*n*=48–97 beta cells/islet, *n*=6–8 mice/group). (**i**) Mean of the coefficients of correlation (mean *r*) for the GCaMP3 fluorescence signal from all possible combinations of single beta cell pairs calculated before and after the application of the glucagon receptor antagonist (*n*=48–97 beta cells/islet, *n*=6–8 mice/group). (**j**) Representative heat maps for every experimental group in the basal state and after administration of the glucagon receptor antagonist, with colour code indicating *r* values for the GCaMP3 fluorescence signal of each beta cell pair. The respective 3D Cartesian maps for the highly correlated beta cells pairs (*r*>0.8) are shown. Data are expressed as individual points. Statistics are based on one-way ANOVA; *p<0.05, **p<0.01, ***p<0.001

Previous studies from our group demonstrate that alpha cell input increases the efficiency of the beta cell response to glucose [13]. Interestingly, an increase in alpha cell mass and glucagon secretion have been described in mouse models exposed to WD-like rich diets [30–32] and in female mice compared with males [33–35]. These data led us to hypothesise that glucagon-secreting alpha cells play a paracrine role in the beta cell [Ca^2+^]_i_ dynamics adaptive mechanism described in our model. To clarify this, female and male mice were transplanted with islets from sex-matched *Ins1*^CreERT2^-*GCaMP3* mice into the ACE and fed for 2 months with CD or WD (Fig. 6a). We focused on the 2 months WD feeding time point as both sexes retained the [Ca^2+^]_i_ dynamics compensatory mechanism described after glucose stimulation.

The alpha cell area of pancreatic islets was increased in both sexes of WD-fed mice, especially in the core of the islets, compared with their respective CD sex-matched groups (Fig. 6b,c), without changes in beta cell area (ESM Fig. 4a). Independently of dietary conditions, female mice presented a greater alpha cell area than male mice. The same observation was made in transplanted islets from the same experimental groups (Fig. 6b,d and ESM Fig. 4b). Accordingly, fasting plasma glucagon levels were increased in WD-fed groups with significantly higher values in females for both dietary conditions (Fig. 6e). As the number of alpha cells and their location changed in a sex-dependent manner in the WD-fed groups during the beta cell compensatory period, we interrogated whether alpha cell input was involved in beta cell [Ca^2+^]_i_ dynamics adaptation. Beta cell [Ca^2+^]_i_ dynamics in the presence of a glucagon receptor antagonist showed a clear reduction of the [Ca^2+^]_i_ signal (Fig. 6f). Both sexes presented an increased periodicity of the slow [Ca^2+^]_i_ oscillations (Fig. 6g) with reduced slow [Ca^2+^]_i_ oscillation amplitude (Fig. 6h) and coordination of the [Ca^2+^]_i_ signal (Fig. 6i,j). These effects were more pronounced in female mice, with less-frequent slow oscillations and reduced [Ca^2+^]_i_ signal coordination, reaching values even below those of the respective CD-fed female group at the same time point of the diet intervention (Fig. 4h,l,m).

### Alpha cell input is required in human islets to adapt [Ca^2+^]_i_ dynamics to high energy demand

**Fig. 7:**
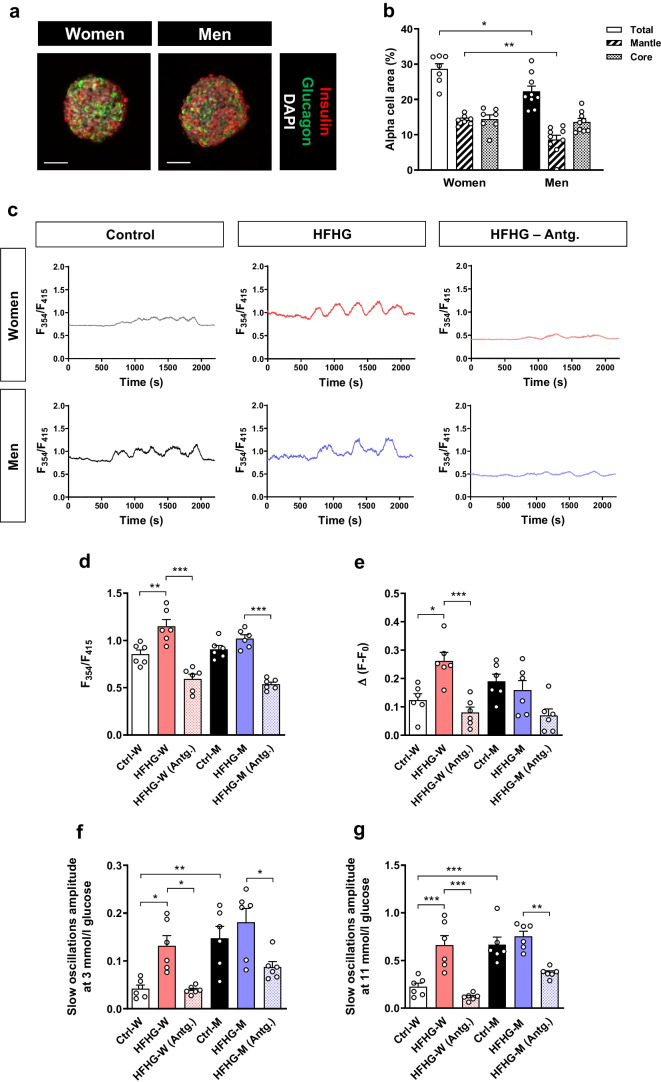
Human islets require alpha cell input to adapt [Ca^2+^]_i_ dynamics to high energy demand. Human islets from cadaveric donors, both women (W) (*n*=3) and men (M) (*n*=3), were cultured under control (Ctrl) or HFHG conditions for 24 h in the presence or absence of the glucagon receptor antagonist L-168,049 (Antg.). (**a**) Representative confocal images of islets from women and men showing immunostaining of beta cells for insulin (red), alpha cells for glucagon (green) and nuclei for DAPI (white). Scale bar, 50 μm. (**b**) Total alpha cell area and differentiating mantle and core regions of islets from women and men (*n*=7–9 islets/group) expressed as % of total islet area. (**c**) Representative [Ca^2+^]_i_ traces of islets from the indicated experimental groups loaded with the fluorescent [Ca^2+^]_i_ indicator Fura-10 and perifused at 3 mmol/l glucose for 500 s followed by 11 mmol/l glucose for 1300 s. Fluorescence values (*F*) were expressed as the ratio of fluorescence at 354 and 415nm (*F*_354_/*F*_415_). (**d**) Fluorescence values for the [Ca^2+^]_i_ levels at 3 mmol/l glucose (*F*_0_) (*n*=6 islets/group). (**e**) Fluorescence increase (Δ*F*−*F*_0_) for the first [Ca^2+^]_i_ peak in response to 11 mmol/l glucose (*n*=6 islets/group). (**f**) Amplitude of slow oscillations during the 3 mmol/l glucose perifusion (*n*=6 islets/group). (**g**) Amplitude of slow oscillations during the 11 mmol/l glucose perifusion (*n*=6 islets/group). Data are presented as individual points. Statistics are based on one-way ANOVA; *p<0.05, **p<0.01, ***p<0.001

We then evaluated in human islets whether [Ca^2+^]_i_ dynamics adapt under high metabolic demand, whether there are sex differences and whether alpha cell input is involved. An increased alpha cell area was observed in islets from women compared with men (Fig. 7a,b). Upon HFHG treatment, basal [Ca^2+^]_i_ dynamics was significantly enhanced in islets from women, when compared with non-treated sex-matched islets, while islets from men only exhibited a non-significant increase (Fig. 7c,d). Sex differences for the glucose response capacity were also observed. HFHG-treated islets from women showed increased amplitude of the first peak after glucose stimulation (Fig. 7c,e), while this difference was not observed in HFHG-treated islets from men. A larger increase in the amplitude of slow oscillations, both at 3 and 11 mmol/l glucose (Fig. 7c,f,g), was also observed in HFHG-treated islets from women compared with men. Thus, islets from women exhibited an increased capacity to adapt [Ca^2+^]_i_ dynamics upon exposure to HFHG. Treatment with glucagon receptor antagonist induced an effect on [Ca^2+^]_i_ dynamics in both sexes, with reduced [Ca^2+^]_i_ levels at 3 mmol/l glucose (Fig. 7c,d), diminished glucose stimulatory peak (Fig. 7c,e) and decreased amplitude of the slow oscillations to values even below those of the sex-matched non-treated islets (Fig. 7c,f,g). This reduction was more evident in islets from women. These data confirm, in humans, the increased capacity of islets from women to adapt to high energy demand and the importance of the alpha cell input for the increased [Ca^2+^]_i_ dynamics adaptive mechanism observed under high metabolic demand.

## Discussion

Sex differences in the risk of developing type 2 diabetes are well known [36]. However, few studies have addressed how these differences play out at the level of beta cell functional adaptation in prediabetes. Here, sex differences in the beta cell functional compensatory response to WD exposure are demonstrated, with the female sex compensating for a longer period of time and the male sex exhibiting early decompensation. Based on the increased proportion of alpha cells observed in the female sex and our findings using a glucagon receptor antagonist, we suggest that glucagon has a predominant role in the protective effect observed in WD-fed mice and in human islets exposed to HFHG in a sex-dependent manner.

Diet intervention models offer suitable conditions with which to study beta cell adaptation processes [3, 5, 37]. In this context, female mice are resistant to developing glucose intolerance due to the protective effect of oestrogens [38, 39] and a different fat distribution compared with males [40]. In males there is a more exacerbated metabolic outcome with faster appearance of beta cell dysfunction resulting in hyperglycaemia [41]. Although glucose tolerance deterioration and hyperglycaemia appeared faster in male mice, we found the same compensatory mechanism in both sexes. Hence, normoglycaemia was maintained by enhanced basal beta cells [Ca^2+^]_i_ dynamics coordination and increased insulin release. Increased basal [Ca^2+^]_i_ dynamics and insulin secretion have previously been described in vivo in a model of diet-induced obesity [5] as an adaptive compensatory mechanism in prediabetes. Here, we identified an increased number of active beta cells with highly coordinated [Ca^2+^]_i_ dynamics in WD-fed mice. These results differ from in vitro studies describing reduced signal coordination upon palmitate treatment [16, 20]. However, our results in vitro in human islets clearly support an increased coordination of the [Ca^2+^]_i_ signal upon HFHG exposure. The observation of more active beta cells in male mice together with the fact that basal [Ca^2+^]_i_ dynamics coordination increases to a lesser extent than in females indicate that beta cells in males constitute a homogeneous population easily reaching their maximum adaptive capacity. In female mice, the progressive increase in beta cell [Ca^2+^]_i_ dynamics indicates the existence of beta cell subpopulations that remain less active for longer periods of time and are only activated under high metabolic demand. The existence of different beta cells populations in terms of functionality has been studied before [42] but, to our knowledge, studies addressing differences between sexes in these subpopulations have not been performed. Because the observed sex differences in beta cell compensation occur at an age range where the female sex is protected by oestrogens, we cannot rule out the possibility that islets from the female sex that are subjected to low oestrogen levels respond in a similar way to those derived from the male sex.

It has been demonstrated that glucose induces a coordinated increase in beta cell [Ca^2+^]_i_ dynamics across the islet in vitro [16, 20], ex vivo [9] and in vivo using the ACE as an islet transplantation site [10]. We describe increased [Ca^2+^]_i_ dynamics coordination after glucose stimulation during the compensatory phase in WD-fed mice. Moreover, longitudinal studies demonstrate that male mice start to decompensate glucose-induced [Ca^2+^]_i_ dynamics earlier than females with an uncoordinated [Ca^2+^]_i_ dynamics response to glucose. A similar drop in glucose-stimulated [Ca^2+^]_i_ dynamics coordination has been described in vitro in islets from mouse models of diabetes [20, 21] and from humans with type 2 diabetes [10], being associated with abnormal insulin secretion oscillatory patterns. In our model this uncoordinated [Ca^2+^]_i_ dynamics response is paralleled by a continuously increased glucose-stimulated insulin secretion. Loss of glucose-stimulated [Ca^2+^]_i_ dynamics coordination linked to uncoordinated insulin secretion has been described as an early sign of diabetes associated with loss of insulin pulsatility and deteriorated insulin sensitivity [43]. Here, we were able to detect those early events prior to losing insulin secretion capacity, by monitoring in vivo beta cell [Ca^2+^]_i_ dynamics.

Previous work from our group demonstrates that glucagon is instrumental for the regulation of glucose homeostasis [13]. Typically, glucagon’s actions in regulating glucose homeostasis are considered in the fasting state [44, 45]. However, glucagon also has an insulinotropic effect in activated beta cells leading to reduced blood glucose concentration [11, 46]. We demonstrate increased alpha cell area and plasma glucagon levels in WD-fed mice during the compensatory phase. These results are in line with other studies showing increased alpha cell area and glucagon secretion in a high-fat-diet-induced obesity model [30–32]. In our model, increased alpha cell proliferation is the most reasonable source for the new alpha cells observed under high metabolic demand. This phenomenon has already been described in other diabetes models [47]. Although beta cell to alpha cell transdifferentiation has also been observed in mouse diabetes models [48] and in humans [49], this only occurs parallel to loss of beta cell identity, which is not the case here.

Different data support a direct role for glucagon in beta cell [Ca^2+^]_i_ dynamics. It has been reported that the alpha cell input is necessary for beta cells to adapt their metabolism to high-fat feeding, mostly by a mechanism involving adequate levels of cAMP [11]. In that work it was claimed that cAMP levels dictate the magnitude of insulin secretion independent of the triggering and amplifying pathways upon glucose stimulation However, other works show how cAMP signalling (mediated by exchange protein activated by cAMP [epac2A] and protein kinase A [PKA]) modulate [Ca^2+^]_i_ levels [50]. That review details the mechanisms by which cAMP might induce a [Ca^2+^]_i_ increase in beta cells. Interestingly, an increase in [Ca^2+^]_i_ levels has been described in glucose-stimulated beta cells in response to glucagon-like peptide-1 (GLP-1) via increased cAMP. This effect seems to involve voltage-dependent Ca^2+^ influx and Ca^2+^-induced Ca^2+^ release from intracellular compartments. The same effect has been observed with exendin-4 (a GLP-1 agonist). Our data with the glucagon receptor antagonist clearly shows an effect of the glucagon input on [Ca^2+^]_i_ oscillation coordination both in mice fed a WD and in human islets cultured under HFHG conditions. The abolished [Ca^2+^]_i_ signalling upon glucagon receptor blockage was stronger in WD-fed female mice and in islets from women. This observation, coinciding with the high proportion of alpha cells observed in female mice and women, support the idea of a higher paracrine effect of glucagon in the female sex. Thus, glucagon has a determinant role in the increased [Ca^2+^]_i_ dynamics coordination adaptive mechanism observed under high metabolic demand, especially in the female sex due to their intrinsic high alpha cell proportion, probably by a mechanism that involves cAMP and Ca^2+^-induced Ca^2+^-release.

Our study has some limitations that should be acknowledged. Further studies are needed to examine the paracrine role of other signalling molecules, such as incretins or neurotransmitters, in beta cell function under glucometabolic challenge. Another limitation of our study is the technical challenges posed by in vivo analysis of basal and glucose-stimulated [Ca^2+^]_i_ dynamics in more than one islet per mouse. There is a lack of evidence in the literature describing equal [Ca^2+^]_i_ responses between islets from the same mice in vivo. However, our results support the notion of similar [Ca^2+^]_i_ patterns between islets from the same experimental groups or sexes.

In summary, we describe sex dimorphism in the functional adaptive capacity of beta cells, assessed for the first time in both sexes in parallel, in vivo and longitudinally at single-cell resolution. We found increased [Ca^2+^]_i_ dynamics coordination to be a mechanism triggering beta cell functional adaptation. In this context, paracrine alpha cell input contributes to the amplification of [Ca^2+^]_i_ dynamics coordination and the increased proportion of alpha cells explain the protective effect in the female sex exposed to metabolic stress. Moreover, an uncoordinated [Ca^2+^]_i_ signal associated with insulin hypersecretion was demonstrated to be an early sign of prediabetes. Our results may have impact on establishing critical time points for predicting beta cell failure and thereby facilitate an early diagnosis during prediabetes, a prerequisite for developing new glucose-lowering therapies to modulate beta cell functional adaptation.

### Supplementary Information

Below is the link to the electronic supplementary material.

ESM (PDF 447 KB)

## Data Availability

The data generated in this study are available from the corresponding authors on reasonable request.

## Abbreviations

ACE: Anterior chamber of the eye
[Ca^2+^]_i_: Cytoplasmic free calcium concentration
CD: Control diet
3D: Three-dimensional
GLP-1: Glucagon-like peptide-1
HFHG: High-fat/high-glucose
WD: Western diet

## References

[1] Weir GC, Bonner-Weir S (2004) Progression to diabetes. Diabetes 53:34–41

[2] Ahrén J, Ahrén B, Wierup N (2010) Increased β-cell volume in mice fed a high-fat diet: a dynamic study over 12 months. Islets 2(6):353–356. 10.4161/isl.2.6.13619 21099337 10.4161/isl.2.6.13619

[3] Gonzalez A, Merino B, Marroquí L et al (2013) Insulin hypersecretion in islets from diet-induced hyperinsulinemic obese female mice is associated with several functional adaptations in individual β-cells. Endocrinology 154(10):3515–3524. 10.1210/en.2013-1424 23867214 10.1210/en.2013-1424

[4] Hull RL, Kodama K, Utzschneider KM, Carr DB, Prigeon RL, Kahn SE (2005) Dietary-fat-induced obesity in mice results in beta cell hyperplasia but not increased insulin release: evidence for specificity of impaired beta cell adaptation. Diabetologia 48(7):1350–1358. 10.1007/s00125-005-1772-9 15937671 10.1007/s00125-005-1772-9

[5] Chen C, Chmelova H, Cohrs CM et al (2016) Alterations in β-cell calcium dynamics and efficacy outweigh islet mass adaptation in compensation of insulin resistance and prediabetes onset. Diabetes 65(9):2676–2685. 10.2337/db15-1718 27207518 10.2337/db15-1718

[6] Irles E, Ñeco P, Lluesma M et al (2015) Enhanced glucose-induced intracellular signaling promotes insulin hypersecretion: pancreatic beta-cell functional adaptations in a model of genetic obesity and prediabetes. Mol Cell Endocrinol 404:46–55. 10.1016/j.mce.2015.01.033 25633666 10.1016/j.mce.2015.01.033

[7] Tramunt B, Smati S, Grandgeorge N et al (2020) Sex differences in metabolic regulation and diabetes susceptibility. Diabetologia 63:453–461. 10.1007/s00125-019-05040-3 31754750 10.1007/s00125-019-05040-3 PMC6997275

[8] MacDonald PE, Joseph JW, Rorsman P (2005) Glucose-sensing mechanisms in pancreatic β-cells. Philos Trans R Soc B: Biol Sci 360(1464):2211–2225. 10.1098/rstb.2005.1762 10.1098/rstb.2005.1762 PMC1569593 16321791

[9] Markovič R, Stožer A, Gosak M, Dolenšek J, Marhl M, Rupnik MS (2015) Progressive glucose stimulation of islet beta cells reveals a transition from segregated to integrated modular functional connectivity patterns. Sci Rep 5:1–10. 10.1038/srep07845 10.1038/srep07845 PMC4297961 25598507

[10] Salem V, Silva LD, Suba K et al (2019) Leader β-cells coordinate Ca2+ dynamics across pancreatic islets in vivo. Nat Metab 1(6):615–629. 10.1038/s42255-019-0075-2 32694805 10.1038/s42255-019-0075-2 PMC7617060

[11] Capozzi ME, Svendsen B, Encisco SE et al (2019) β Cell tone is defined by proglucagon peptides through cAMP signaling. JCI Insight 4(5):e126742. 10.1172/jci.insight.126742 30720465 10.1172/jci.insight.126742 PMC6483521

[12] Svendsen B, Larsen O, Gabe MBN et al (2018) Insulin secretion depends on intra-islet glucagon signaling. Cell Rep 25(5):1127-1134.e2. 10.1016/j.celrep.2018.10.018 30380405 10.1016/j.celrep.2018.10.018

[13] Rodriguez-Diaz R, Molano RD, Weitz JR et al (2018) Paracrine interactions within the pancreatic islet determine the glycemic set point. Cell Metab 27(3):549-558.e4. 10.1016/j.cmet.2018.01.015 29514065 10.1016/j.cmet.2018.01.015 PMC5872154

[14] Westacott MJ, Ludin NWF, Benninger RKP (2017) Spatially organized β-cell subpopulations control electrical dynamics across islets of Langerhans. Biophys J 113(5):1093–1108. 10.1016/j.bpj.2017.07.021 28877492 10.1016/j.bpj.2017.07.021 PMC5658715

[15] Gustavsson N, Larsson-Nyrén G, Lindström P (2005) Pancreatic β cells from db/db mice show cell-specific [Ca 2+]i and NADH responses to glucose but not to α-ketoisocaproic acid. Pancreas 31(3):242–250. 10.1097/01.mpa.0000175891.58918.c8 16163056 10.1097/01.mpa.0000175891.58918.c8

[16] Johnston NR, Mitchell RK, Haythorne E et al (2016) Beta cell hubs dictate pancreatic islet responses to glucose. Cell Metab 24(3):389–401. 10.1016/j.cmet.2016.06.020 27452146 10.1016/j.cmet.2016.06.020 PMC5031557

[17] Colsoul B, Jacobs G, Philippaert K et al (2014) Insulin downregulates the expression of the Ca2+-activated nonselective cation channel TRPM5 in pancreatic islets from leptin-deficient mouse models. Pflugers Arch 466(3):611–621. 10.1007/s00424-013-1389-7 24221356 10.1007/s00424-013-1389-7 PMC3928505

[18] Reinbothe TM, Alkayyali S, Ahlqvist E et al (2013) The human L-type calcium channel Cav13 regulates insulin release and polymorphisms in CACNA1D associate with type 2 diabetes. Diabetologia 56(2):340–349. 10.1007/s00125-012-2758-z 23229155 10.1007/s00125-012-2758-z

[19] Benninger RKP, Piston DW (2014) Cellular communication and heterogeneity in pancreatic islet insulin secretion dynamics. Trends Endocrinol Metab 25(8):399–406. 10.1016/j.tem.2014.02.005 24679927 10.1016/j.tem.2014.02.005 PMC4112137

[20] Hodson DJ, Mitchell RK, Bellomo EA et al (2013) Lipotoxicity disrupts incretin-regulated human β cell connectivity. J Clin Investig 123(10):4182–4194. 10.1172/JCI68459 24018562 10.1172/JCI68459 PMC4382273

[21] Ravier M, Sehlin J, Henquin J (2002) Disorganization of cytoplasmic Ca2+ oscillations and pulsatile insulin secretion in islets from ob/ob mice. Diabetologia 45(8):1154–1163. 10.1007/s00125-002-0883-9 12189446 10.1007/s00125-002-0883-9

[22] Idevall-Hagren O, Tengholm A (2020) Metabolic regulation of calcium signaling in beta cells. Semin Cell Dev Biol 103:20–30. 10.1016/j.semcdb.2020.01.008 32085965 10.1016/j.semcdb.2020.01.008

[23] Zhang M, Fendler B, Peercy B et al (2008) Long lasting synchronization of calcium oscillations by cholinergic stimulation in isolated pancreatic islets. Biophys J 95(10):4676–4688. 10.1529/biophysj.107.125088 18708464 10.1529/biophysj.107.125088 PMC2576377

[24] Jacob S, Köhler M, Tröster P et al (2020) In vivo Ca2+ dynamics in single pancreatic β cells. FASEB J 34(1):945–959. 10.1096/fj.201901302RR 31914664 10.1096/fj.201901302RR

[25] Thorens B, Tarussio D, Maestro MA, Rovira M, Heikkilä E, Ferrer J (2015) Ins1 Cre knock-in mice for beta cell-specific gene recombination. Diabetologia 58(3):558–565. 10.1007/s00125-014-3468-5 25500700 10.1007/s00125-014-3468-5 PMC4320308

[26] Zariwala HA, Borghuis BG, Hoogland TM et al (2012) A Cre-dependent GCaMP3 reporter mouse for neuronal imaging in vivo. J Neurosci 32(9):3131–3141. 10.1523/JNEUROSCI.4469-11.2012 22378886 10.1523/JNEUROSCI.4469-11.2012 PMC3315707

[27] Arrojo e Drigo R, Jacob S, García-Prieto C et al (2019) Structural basis for delta cell paracrine regulation in pancreatic islets. Nat Commun 10(1):3700. 10.1038/s41467-019-11517-x 31420552 10.1038/s41467-019-11517-x PMC6697679

[28] Li L, Trifunovic A, Köhler M et al (2014) Defects in β-cell Ca2+ dynamics in age-induced diabetes. Diabetes 63(12):4100–4114. 10.2337/db13-1855 24985350 10.2337/db13-1855

[29] Bosco D, Armanet M, Morel P et al (2010) Unique arrangement of α- and β-cells in human islets of Langerhans. Diabetes 59(5):1202–1210. 10.2337/db09-1177 20185817 10.2337/db09-1177 PMC2857900

[30] Lee YS, Jun HS (2018) Glucagon-like peptide-1 receptor agonist and glucagon increase glucose-stimulated insulin secretion in beta cells via distinct adenylyl cyclases. Int J Med Sci 15(6):603–609. 10.7150/ijms.24492 29725251 10.7150/ijms.24492 PMC5930462

[31] Ellingsgaard H, Ehses JA, Hammar EB et al (2008) Interleukin-6 regulates pancreatic α-cell mass expansion. Proc Natl Acad Sci U S A 105(35):13163–13168. 10.1073/pnas.0801059105 18719127 10.1073/pnas.0801059105 PMC2529061

[32] Kellard JA, Rorsman NJG, Hill TG et al (2020) Reduced somatostatin signalling leads to hypersecretion of glucagon in mice fed a high-fat diet. Mol Metab 40:101021. 10.1016/j.molmet.2020.101021 32446876 10.1016/j.molmet.2020.101021 PMC7322681

[33] Bonnevie-Nielsen V (1980) Experimental diets affect pancreatic insulin and glucagon differently in male and female mice. Metabolism 29(4):386–91. 10.1016/0026-0495(80)90014-1 6990176 10.1016/0026-0495(80)90014-1

[34] Bonnevie-Nielsen V (1982) Different effects of high glucose and high fat diet on pancreatic insulin and glucagon in female and male mice. Diabete Metab 8(4):271–277 6761182

[35] Karlsson S, Scheurink AJ, Ahrén B (2002) Gender difference in the glucagon response to glucopenic stress in mice. Am J Physiol Regul Integr Comp Physiol 282(1):R281-8. 10.1152/ajpregu.2002.282.1.R281 11742849 10.1152/ajpregu.2002.282.1.R281

[36] Tramunt B, Smati S, Grandgeorge N et al (2020) Sex differences in metabolic regulation and diabetes susceptibility. Diabetologia 63:453–461. 10.1007/s00125-019-05040-3 31754750 10.1007/s00125-019-05040-3 PMC6997275

[37] Sone H, Kagawa Y (2005) Pancreatic beta cell senescence contributes to the pathogenesis of type 2 diabetes in high-fat diet-induced diabetic mice. Diabetologia 48(1):58–67. 10.1007/s00125-004-1605-2 15624098 10.1007/s00125-004-1605-2

[38] Stubbins RE, Holcomb VB, Hong J, Núñez NP (2012) Estrogen modulates abdominal adiposity and protects female mice from obesity and impaired glucose tolerance. Eur J Nutr 51(7):861–870. 10.1007/s00394-011-0266-4 22042005 10.1007/s00394-011-0266-4

[39] Riant E, Waget A, Cogo H, Arnal JF, Burcelin R, Gourdy P (2009) Estrogens protect against high-fat diet-induced insulin resistance and glucose intolerance in mice. Endocrinology 150(5):2109–2117. 10.1210/en.2008-0971 19164473 10.1210/en.2008-0971

[40] Comitato R, Saba A, Turrini A, Arganini C, Virgili F (2015) Sex hormones and macronutrient metabolism. Crit Rev Food Sci Nutr 55(2):227–241. 10.1080/10408398.2011.651177 24915409 10.1080/10408398.2011.651177 PMC4151815

[41] Nishikawa S, Yasoshima A, Doi K, Nakayama H, Uetsuka K (2007) Diet-induced obesity in C57BL / 6J and BALB / cA mice. Obesity 56:263–272 10.1538/expanim.56.263 17660680

[42] Dorrell C, Schug J, Canaday PS et al (2016) Human islets contain four distinct subtypes of β cells. Nat Commun 11(7):11756. 10.1038/ncomms11756 10.1038/ncomms11756 PMC4942571 27399229

[43] Satin LS, Butler PC, Ha J, Sherman AS (2015) Pulsatile insulin secretion, impaired glucose tolerance and type 2 diabetes. Mol Aspects Med 42:61–77. 10.1016/j.mam.2015.01.003 25637831 10.1016/j.mam.2015.01.003 PMC4696552

[44] Gromada J, Franklin I, Wollheim CB (2007) Α-cells of the endocrine pancreas: 35 years of research but the enigma remains. Endocr Rev 28(1):84–116. 10.1210/er.2006-0007 17261637 10.1210/er.2006-0007

[45] Jiang G, Zhang BB (2003) Glucagon and regulation of glucose metabolism. Am J Physiol Endocrinol Metab 284(4):E671-8. 10.1152/ajpendo.00492.2002 12626323 10.1152/ajpendo.00492.2002

[46] Capozzi ME, Wait JB, Koech J et al (2019) Glucagon lowers glycemia when β cells are active. JCI Insight 5(16):e129954. 10.1172/jci.insight.129954 31335319 10.1172/jci.insight.129954 PMC6777806

[47] Liu Z, Kim W, Chen Z et al (2011) Insulin and glucagon regulate pancreatic α-cell proliferation. PLoS One 25(6):e16096. 10.1371/journal.pone.0016096 10.1371/journal.pone.0016096 PMC3026810 21283589

[48] Tanday N, Flatt PR, Irwin N, Moffett RC (2020) Liraglutide and sitagliptin counter beta- to alpha-cell transdifferentiation in diabetes. J Endocrinol 245(1):53–64. 10.1530/JOE-19-0451 31977315 10.1530/JOE-19-0451

[49] Spijker HS, Ravelli RB, Mommaas-Kienhuis AM et al (2013) Conversion of mature human β-cells into glucagon-producing α-cells. Diabetes 62(7):2471–2480. 10.2337/db12-1001 23569174 10.2337/db12-1001 PMC3712074

[50] Stožer A, Paradiž Leitgeb E, Pohorec V et al (2021) The role of cAMP in beta cell stimulus-secretion and intercellular coupling. Cells 10(7):1658. 10.3390/cells10071658 34359828 10.3390/cells10071658 PMC8304079

