# Supplementary material for: Sex-dependent intra-islet structural rearrangements affecting alpha-to-beta cell interactions lead to adaptive enhancements of Ca2+ dynamics in prediabetic beta cells
Source: Diabetologia. 2024 May 30;67(8):1663–82. doi: 10.1007/s00125-024-06173-w (PMC11343800; doi:10.1007/s00125-024-06173-w)
Supplement: Supplementary file 1 — ESM (PDF 447 KB) [file 125_2024_6173_MOESM1_ESM.pdf]

Electronic Supplementary Material

Sex-dependent intra-islet structural rearrangements affecting alpha-to-beta cell interactions lead to adaptive enhancements of Ca^{2+} dynamics in prediabetic beta cells

ESM Methods

In vivo beta cell $[Ca^{2+}]_i$ imaging

In vivo beta cell $[Ca^{2+}]_i$ dynamics imaging was performed 3 months after transplantation and once a month until the end of the diet treatment. For longitudinal studies the same islet was imaged over time. 4 h fasted mice were anesthetized by intraperitoneal injection of fluanisone/fentanyl/midazolam (20/0.6/10 mg/kg). Islets were imaged for 10 min in the non-stimulated state followed by 40 min after glucose injection through the tail vein (0.4 g/kg). GCaMP3 fluorescence was collected in an iXon/Ultra888/EMCCD camera (Andor) installed on top of a Leica TCS-SP5II microscope. Images were recorded using a 25x objective controlled by a piezo element managed by LabView software to perform 60 images every 2 s with 4 µm z-stacks thickness between images. After 10 min of imaging in the non-stimulatory period the glucagon-receptor antagonist L-168,049 (4 mmol/l, Tocris) was topically applied to the eye and, 5 min post topical application, islets were imaged for 20 min.

$[Ca^{2+}]_i$ image processing and analysis

In vivo beta cell $[Ca^{2+}]_i$ traces were extracted from GCaMP3 fluorescence images processed using custom-made MATLAB scripts [24,27]. Single beta cells were automatically identified based on their temporal and spatial GCaMP3 fluorescence profile. We defined as basal GCaMP3 fluorescence the mean of the lowest 25% fluorescence intensity values prior to glucose stimulation. The entire GCaMP3 fluorescence recording for every single-cell was normalized against their basal GCaMP3 signal and expressed as fold increase over the baseline ($\Delta F/F_0$). Normalized GCaMP3 traces were used to detect $[Ca^{2+}]_i$ peaks using a peak-finding function adjusted to identify peaks 1x higher than the standard deviation of the entire GCaMP3 signal [24,27]. The percentage of cells presenting significant $[Ca^{2+}]_i$ spiking activity was calculated by comparison of the amplitude and location of peaks detected in single beta cells against the whole islet GCaMP3 signal, measured as the mean of the signal of all single beta cells detected. GCaMP3 fluorescence signal was also analysed using power spectral analysis by a custom-made MATLAB script adapted to differentiate between fast (6-60 s) and slow oscillations (60-600 s) [28]. The percentage of glucose responding beta cells was analysed considering those cells in which the GCaMP3 fluorescence intensity of the first peak after glucose stimulation was 2x higher than the standard deviation of the basal GCaMP3 fluorescence. The term $[Ca^{2+}]_i$ dynamics was defined as all changes in $[Ca^{2+}]_i$ including fast and slow oscillations, peak values as well as plateau values.

In vitro $[Ca^{2+}]_i$ measurements

Human islets were loaded for 1h with Fura-10 (2 µmol/l; AATBioquest) in a buffered solution (pH 7.4; 125 mmol/l NaCl, 5.9 mmol/l KCl, 2.56 mmol/l $CaCl_2$, 1.2 mmol/L $MgCl_2$, 25 mmol/l HEPES and 0.1% BSA) supplemented with 3 mmol/l glucose. $[Ca^{2+}]_i$ signal was recorded using an epifluorescence microscope connected to a Fluorolog spectrofluorometer. Islets were perifused at 37° C with the buffered solution at 3 mmol/l or 11 mmol/l glucose in the presence or absence of the glucagon-receptor antagonist L-168,049 (50 nmol/l). Fluorescence values (F) were expressed as the ratio of fluorescence at 354 and 415nm (F_{354}/F_{415}). $[Ca^{2+}]_i$ levels at 3 mmol/l glucose (F_0) were analysed as mean of the fluorescence values at 3 mmol/l glucose. Glucose-stimulation was assessed by subtracting F_0 from the first stimulatory $[Ca^{2+}]_i$ peak after perifusion at 11 mmol/l glucose (ΔF-F_0). Slow $[Ca^{2+}]_i$ oscillations were analysed with regard to amplitude using a custom-made MATLAB script.

Immunostaining and islet morphometric analysis

Pancreases, explanted islets from the ACE and islets in culture were fixed in 4% paraformaldehyde. Pancreas sections and islets were permeabilized in 0.5% Triton and blocked with 10% FBS in PBS for 1 h at room temperature, followed by incubation at 4ºC overnight with guinea pig anti-insulin (1:1000; Dako) and mouse anti-glucagon (1:500; Sigma-Aldrich) primary antibodies diluted in blocking solution and with the corresponding secondary antibodies (goat anti-guinea pig AlexaFluor 546 [1:500; ThermoFisher] and goat anti-mouse AlexaFluor 488 [1:500; ThermoFisher]) diluted in blocking solution for 1 h at room temperature. Sections and islets were mounted in ProLong Gold with DAPI (ThermoFisher) and visualized using a Leica TCS-SP5II microscope. Three sections of every pancreas were acquired and images were analysed using ImageJ/Fiji. Insulin and glucagon-positive areas were determined for every islet and expressed in relation to the total islet area. Mantel and core areas of the islets were analysed differentiating the area 20 µm from the external islet perimeter for mantel area quantification and the rest of the islet for core quantification [29].

ESM Table

Sex	Age (years)	BMI (kg / m^2)	HbA1c mmol/mol (%)	History of diabetes
Woman	36	18.9	32 (5.1%)	Healthy
Woman	58	22.5	38 (5.6%)	Healthy
Woman	63	22.0	37 (5.5%)	Healthy
Man	75	23.8	33 (5.2%)	Healthy
Man	62	22.2	36 (5.4%)	Healthy
Man	65	22.3	38 (5.6%)	Healthy

ESM Table 1 Human islet donor details. Information about sex, age, body mass index (BMI) and glycated haemoglobin (HbA1c) of each human islet donor. Based on BMI and HbA1c data, donors were classified as healthy individuals without diabetes.

ESM Figures

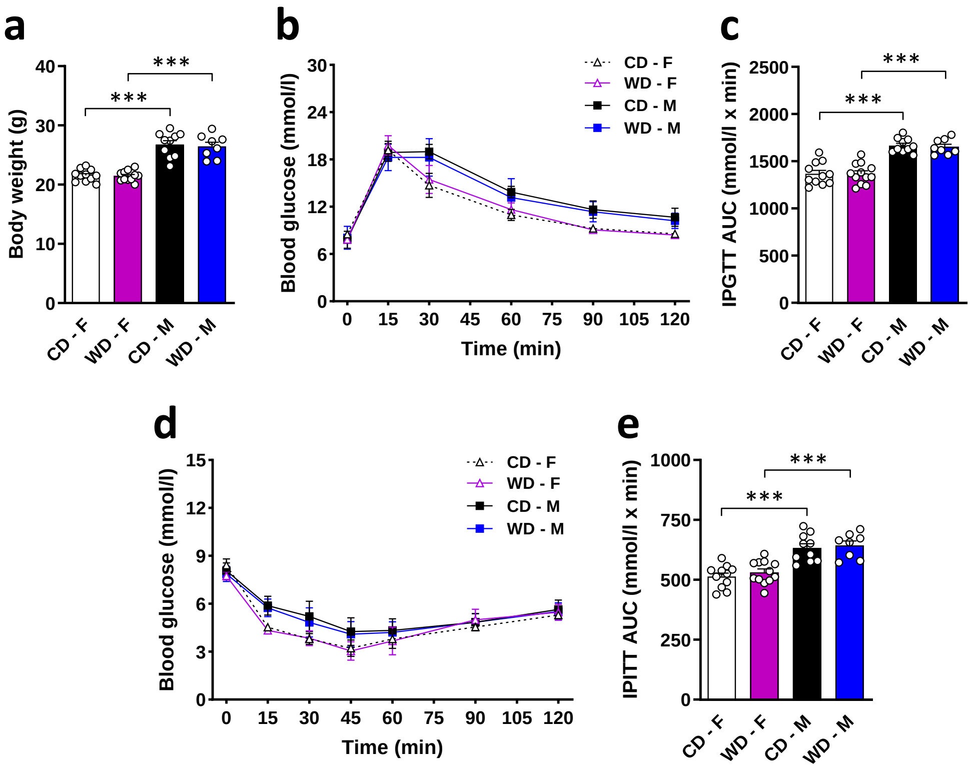

ESM Fig. 1 Metabolic features in sex-matched groups prior to diet assignment. (**a**) Body weight of C57Bl/6J female (F) and male (M) mice fed a control diet (CD) and prior to western diet (WD) assignment (*n*=8-12 mice/group). (**b**, **c**) IPGTT blood glucose levels (**b**) and corresponding AUC (**c**) in the same mice groups as **a**. (**d**, **e**) IPITT blood glucose levels (**d**) and corresponding AUC (**e**) assessed in the same mice groups as **a**. Data are presented as individual points (**a**, **c**, **e**) or mean ± SEM (**b**, **d**). Statistics are based on one-way ANOVA (**a**, **c**, **e**; $^{***}p$ <0.001) or two-way ANOVA (**b**, **d**).

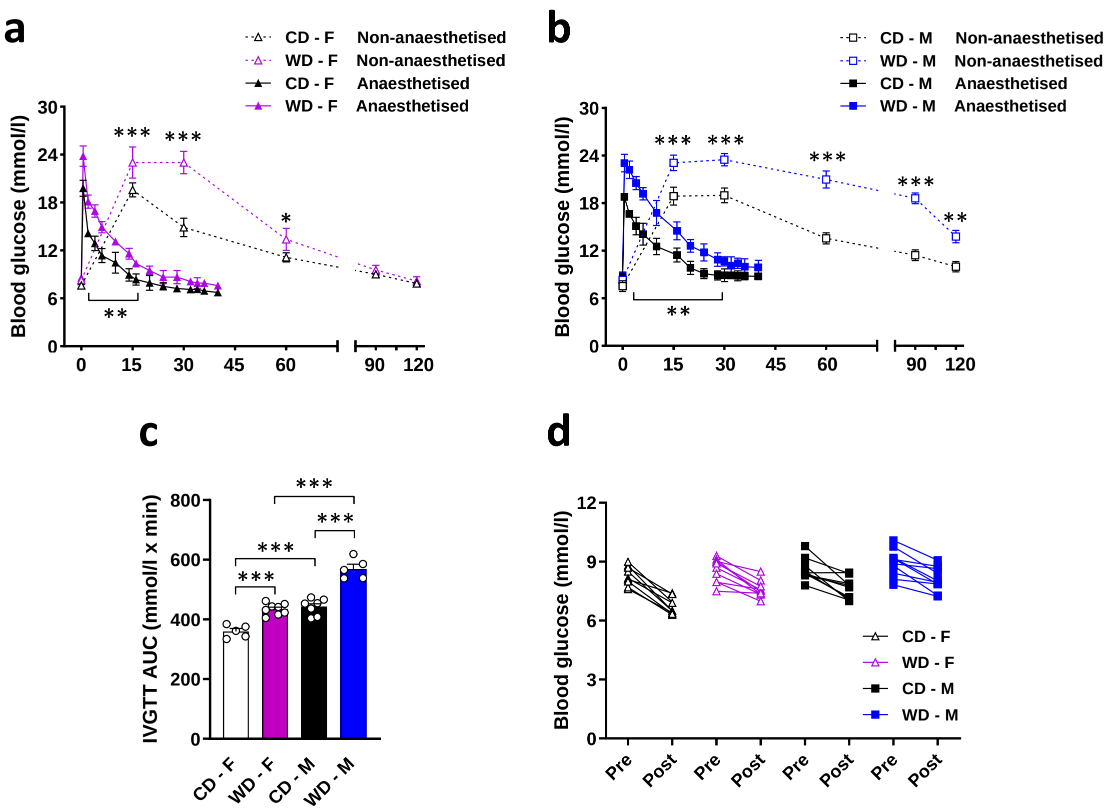

ESM Fig. 2 Glucose homeostasis under anaesthesia. (**a**) IPGTT blood glucose levels in female mice fed a control diet (CD) or western diet (WD) for 1 month in non – anaesthetised mice (discontinuous lines) (*n*=8-10 mice/group) and IVGTT blood glucose levels in female mice fed a CD or WD for 1 month and anaesthetised under the same conditions as those applied for the in vivo beta cell $[Ca^{2+}]_i$ dynamics imaging experiments (continuous lines) (*n*=5-8 mice/group). (**b**) IPGTT blood glucose levels in male mice fed a CD or WD for 1 month in non – anaesthetised mice (discontinuous lines) (*n*=8-10 mice/group) and IVGTT blood glucose levels in male mice fed a CD or WD for 1 month and anaesthetised under the same conditions as those applied for the in vivo beta cell $[Ca^{2+}]_i$ dynamics imaging experiments (continuous lines) (*n*=5-7 mice/group). (**c**) AUC corresponding to the IVGTT blood glucose levels in **a** and **b**. (**d**) Blood glucose levels measured in anaesthetised mice before (Pre) and after (Post) in vivo imaging of *Ins1*^{CreERT2}-*GCaMP3* islets transplanted into the anterior chamber of the eye (ACE) of female (F) and male (M) mice fed a CD or WD diet for 1 month (*n*=10 mice/group). Data are presented as mean ± SEM (**a**, **b**) or individual points (**c**, **d**). Statistics are based on two-way ANOVA (**a**, **b**; $^{*}p$ <0.05, $^{**}p$ <0.01, $^{***}p$ <0.001) or one-way ANOVA (**c**; $^{***}p$ <0.001).

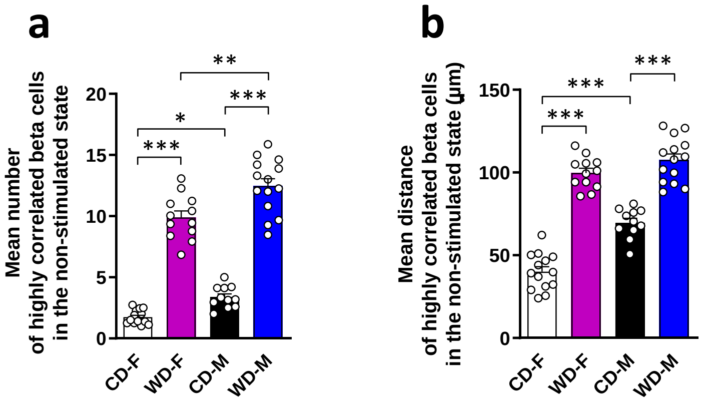

ESM Fig. 3 WD induces complex highly correlated beta cell networks in terms of $[Ca^{2+}]_i$ dynamics. (**a**) Mean number of highly correlated beta cell pairs (r>0.8) for basal (10 min) GCaMP3 fluorescence of *Ins1*^{CreERT2}-*GCaMP3* islet transplanted in the ACE of female (F) and male (M) mice fed a CD or for 1 month on WD (n=11-14 mice/group). (**b**) Mean distance between beta cell pairs calculated from the *x*, *y*, *z* position in 3D of highly correlated beta cell pairs (r>0.8) (n=11-14 mice/group) in the same mice groups and conditions as **a**. Data are presented as individual points. Statistics are based on one-way ANOVA; * p <0.05, ** p <0.01, *** p <0.001.

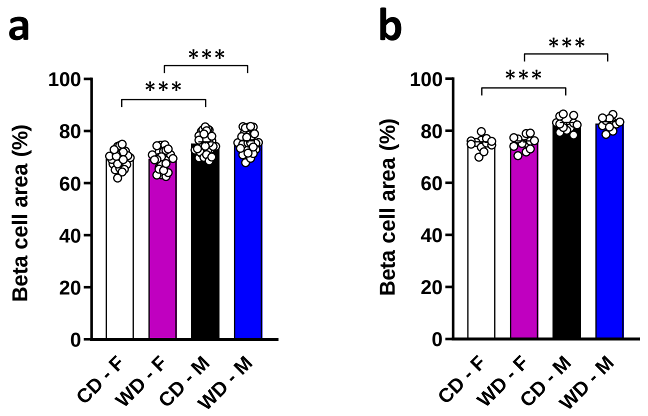

ESM Fig. 4 Beta cell area is maintained under WD feeding. (**a**) Beta cell area measured in all islets detected in three different sections per pancreas (n=3 mice/group) from female (F) and male (M) mice fed a CD or for 2 months on WD. (**b**) Beta cell area of islets explanted from the ACE (n=11-13 islets/group) from the same experimental groups as in **a**. Data are presented as individual points. Statistics are based on one-way ANOVA; $^{***}p < 0.001$.

Checklist for reporting human islet preparations used in research

Adapted from Hart NJ, Powers AC (2018) Progress, challenges, and suggestions for using human islets to understand islet biology and human diabetes. Diabetologia https://doi.org/10.1007/s00125-018-4772-2

Islet preparation	1	2	3	4	5	6	7	8[a]
**MANDATORY INFORMATION**								
Unique identifier	H2498	H2617	H2651	H2539	H2610	H2613		
Donor age (years)	36	58	63	75	62	65		
Donor sex (M/F)	F	F	F	M	M	M		
Donor BMI (kg/m^2)	18.9	22.5	22.0	23.8	22.2	22.3		
Donor HbA_{1c} or other measure of blood glucose control mmol/mol (%)	32 (5.1%)	38 (5.6%)	37 (5.5%)	33 (5.2%)	36 (5.4%)	38 (5.6%)		
Origin/source of islets[b]	Uppsala	Uppsala	Uppsala	Uppsala	Uppsala	Uppsala		
Islet isolation centre	Uppsala	Uppsala	Uppsala	Uppsala	Uppsala	Uppsala		
Donor history of diabetes? Please select yes/no from drop down list	No	No	No	No	No	No		
**If Yes, complete the next two lines if this information is available**								
Diabetes duration (years)								
Glucose-lowering therapy at time of death[c]								
**RECOMMENDED INFORMATION**								
Donor cause of death								
Warm ischaemia time (h)								
Cold ischaemia time (h)								

Estimated purity (%)								
Estimated viability (%)								
Total culture time (h)[d]								
Glucose-stimulated insulin secretion or other functional measurement[e]								
Handpicked to purity? Please select yes/no from drop down list								
Additional notes								

[a]If you have used more than eight islet preparations, please complete additional forms as necessary
[b]For example, IIDP, ECIT, Alberta IsletCore
[c]Please specify the therapy/therapies
[d]Time of islet culture at the isolation centre, during shipment and at the receiving laboratory
[e]Please specify the test and the results
